# 1292. Evaluation of Synergy with Piperacillin/Tazobactam plus Meropenem Against Carbapenemase-Producing *Klebsiella pneumoniae* and *Enterobacter cloacae* Using ETEST

**DOI:** 10.1093/ofid/ofab466.1484

**Published:** 2021-12-04

**Authors:** Deborah S Ashcraft, Royanne H Vortisch, George A Pankey

**Affiliations:** Ochsner Clinic Foundation, New Orleans, Louisiana

## Abstract

**Background:**

Carbapenem-resistant *Enterobacterales* are considered an urgent threat for patients in healthcare facilities, causing infections with significant morbidity and mortality. Most isolates are multidrug resistant with limited treatment options, so combination therapy is an alternative. Recently, synergy with piperacillin/tazobactam (P/T) + meropenem (MP) was demonstrated against 7/10 (70%) KPC-producing *Escherichia coli* and 9/10 (90%) OXA-48-producing *K. pneumoniae* using time-kill assay (Lawandi et al, 2021). The aim of the present study was to further evaluate the combination of P/T + MP against KPC-producing *Enterobacter cloacae*, in addition to OXA-producing *K. pneumoniae* using our rapid ETEST MIC:MIC synergy method.

**Methods:**

14 carbapenemase-producing isolates: 7 OXA-48-like *K. pneumoniae* (1 OXA-48, 4 OXA-181, 2 OXA-232) and 7 KPC-producing *E. cloacae* (1 KPC-2, 4 KPC-3, 1 KPC-4, 1 KPC-6) were obtained from the CDC and FDA Antibiotic Resistance Isolate Bank. ETEST MICs for P/T and MP and our ETEST synergy method were performed in triplicate for each isolate. The summation fractional inhibitory concentration was calculated, and the mean value was interpreted as: < 0.5 synergy; > 0.5-1 additivity; > 1-4 indifference; and > 4 antagonism.

**Results:**

MICs (µg/mL) ranged: MP, 0.5 to > 32 (14% susceptible) and P/T, 96/4 to > 256/4 (all resistant). The combination of P/T + MP showed synergy (3) or additivity (2) against 5/7 (71%) OXA-producing *K. pneumoniae* and synergy (6) or additivity (1) against all 7 KPC-producing *E. cloacae*. No antagonism was detected.

**Conclusion:**

Using our ETEST MIC:MIC method, the combination of P/T + MP demonstrated synergy or additivity in 5/7 OXA-producing *K. pneumoniae* and 7/7 KPC-producing *E. cloacae,* similar to previously published findings showing synergy in 7/10 KPC-producing *E. coli* and 9/10 OXA-48-producing *K. pneumoniae* using time-kill assay. Our ETEST synergy method is simple to use and should be evaluated more extensively. Regardless of the method used, results may or may not correlate in an *in vivo* setting. *In vivo* studies are needed.

**Disclosures:**

**All Authors**: No reported disclosures

